# Supplementary material for: Stable inheritance of the Streptomyces linear plasmid SCP1 by dual ParABS partition systems
Source: Nucleic Acids Res. 2026 Jun 8;54(11):gkag593. doi: 10.1093/nar/gkag593 (PMC13245405; doi:10.1093/nar/gkag593)
Supplement: gkag593_Supplemental_Files [file gkag593_supplemental_files.zip › McPhillips_SCP1_figures_SUPPLEMENTARY.pdf]

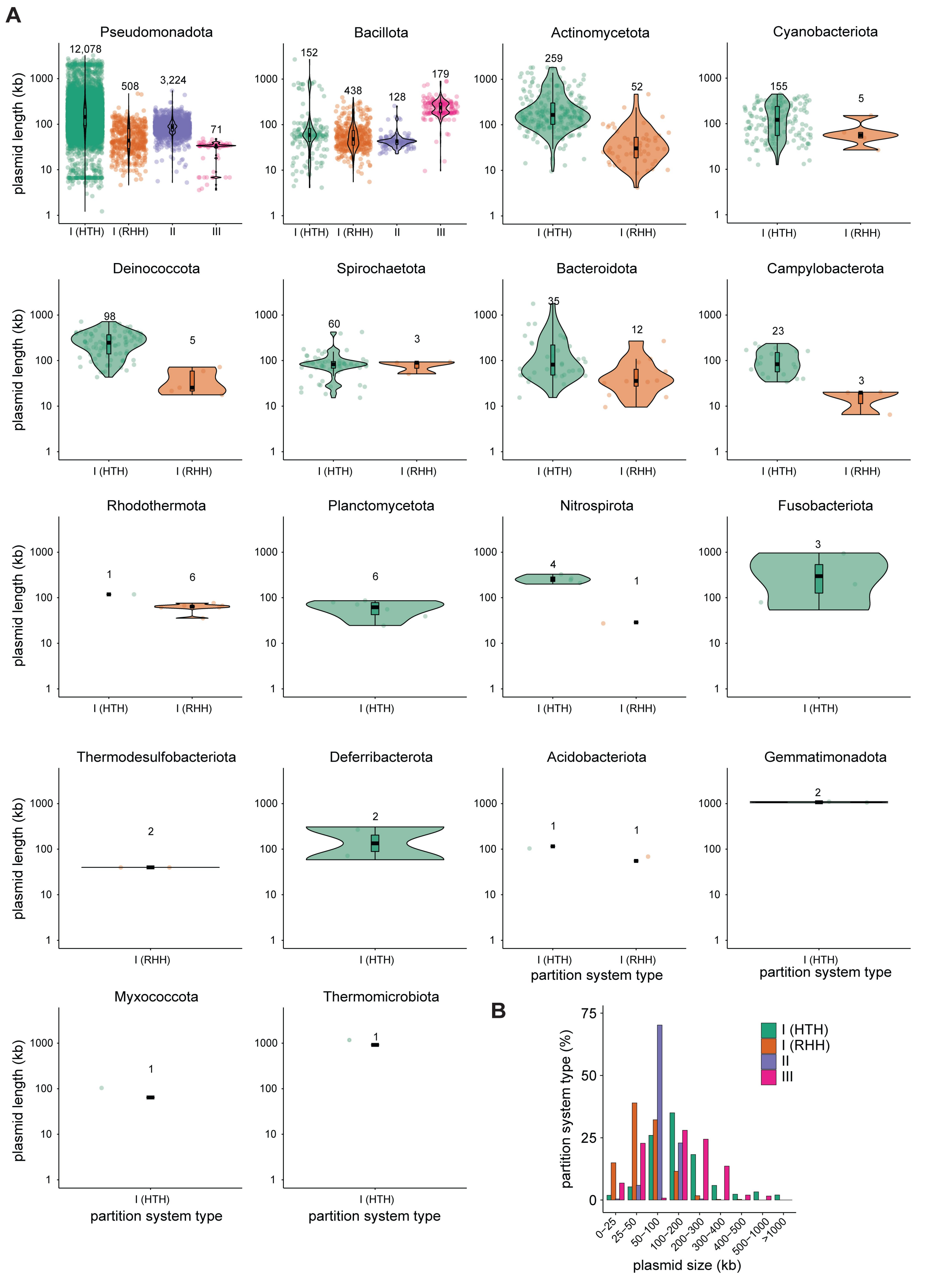

Supplementary Figure S1. Type-I partition systems were predicted to have the broadest taxonomic distribution. **(A)** Distribution of partition system types across PLSDB plasmids with a single partition system. Plasmids were categorized according to bacterial phyla they were isolated from. **(B)** Percentage of each partition system type on PLSDB plasmids with a single partition system across plasmid size bins.

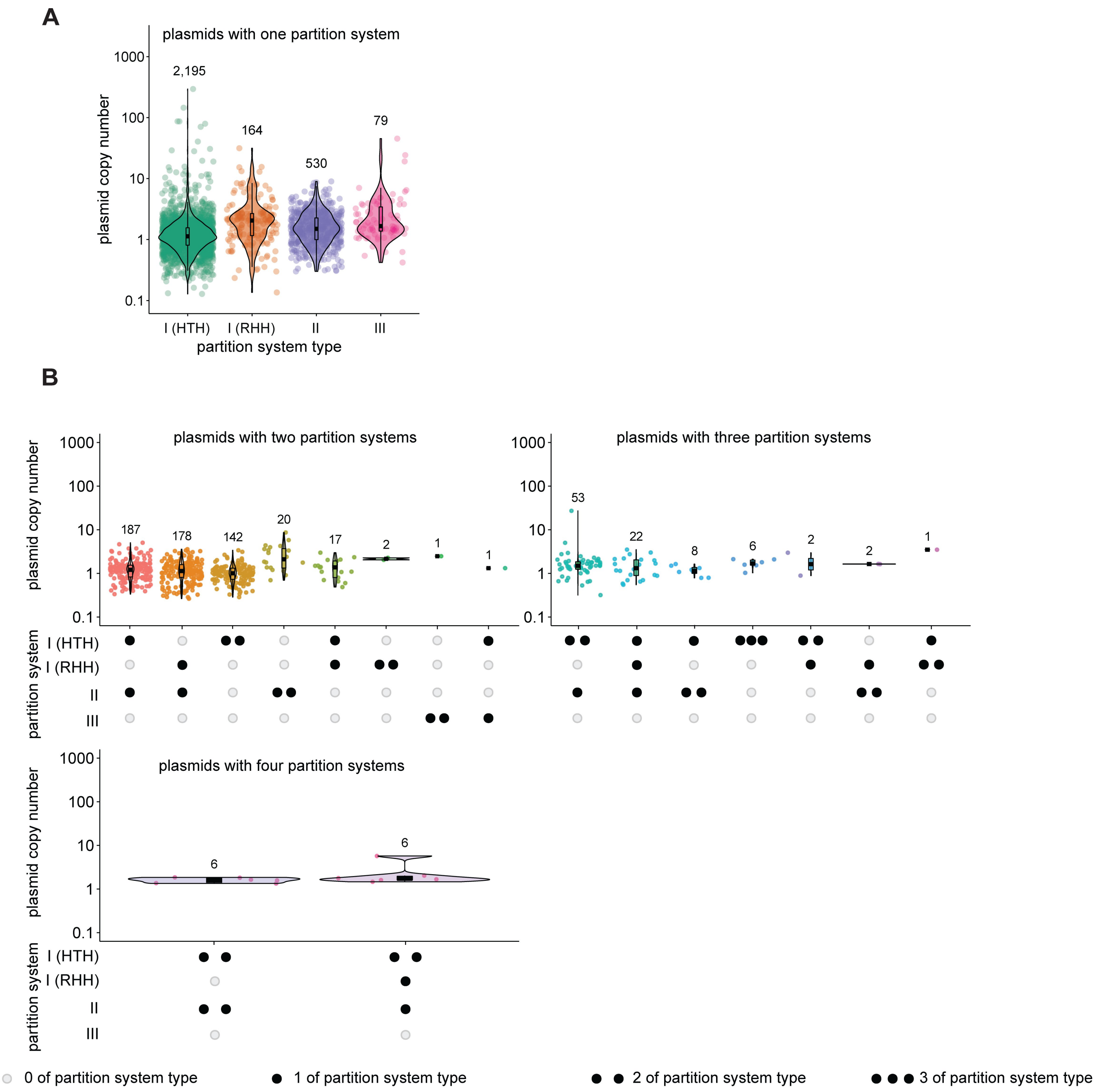

Supplementary Figure S2. The majority of plasmids encoding partition systems are low copy number. **(A)** Plasmids with a single partition system tend to be low copy number with a median plasmid copy number (PCN) of between ~1-2 copies per chromosome. **(B)** Plasmids with multiple partition system also tend to be low copy number with a median PCN between ~1-3.5 copies per chromosome. Filled circles (single or multiple) indicate the presence of one or more corresponding partition system type(s), while unfilled circles indicate the absence of such partition system type(s). PCN data is from Maddamsetti *et al.*, 2025.

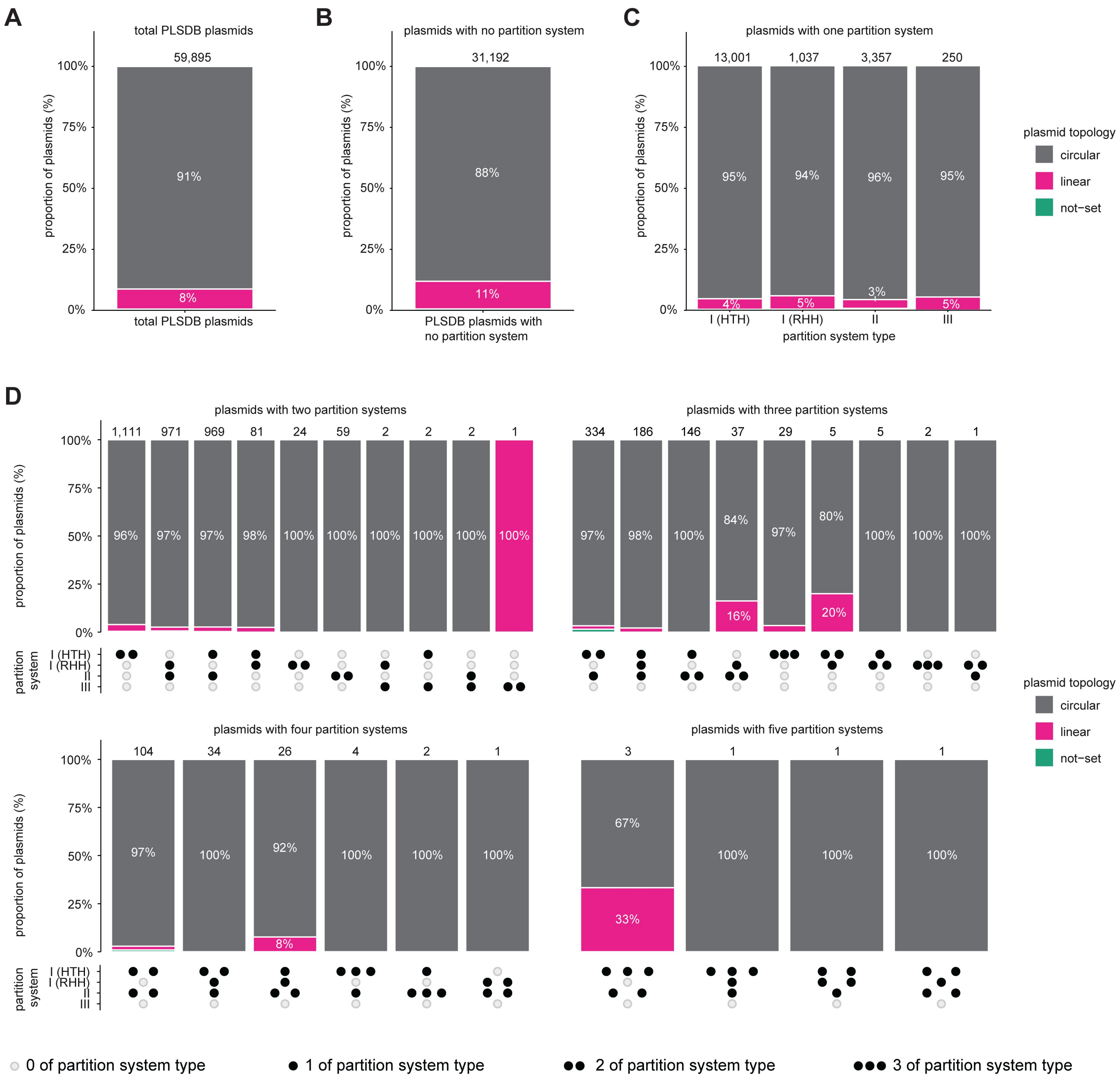

Supplementary Figure S3. Circular plasmids are the dominant plasmid topology across the PLSDB. Plasmid topology was classified as circular (grey), linear (pink) or unknown/undetermined (green). **(A)** All 59,895 PLSDB plasmids classified by topology: circular (54,646 plasmids/~91% of the PLSDB), linear (4,941 plasmids/~8% of the PLSDB) or unknown (308 plasmids/<1% of the PLSDB) topologies. **(B)** The 31,192 PLSDB plasmids that were not predicted to encode a partition system classified by topology: circular (27,476 plasmids/~88% of no partition system plasmids), linear (3,529 plasmids/~11% of no partition system plasmids) or unknown (187 plasmids/<1% of no partition system plasmids) topologies. **(C)** The 17,645 PLSDB plasmids predicted to encode one partition system separated based on their partition system type and whether they are predicted to have a circular (16,842/~95% of single partition system plasmids), linear (732/~4% of single partition system plasmids) or unknown (71/<1% of single partition system plasmids) topologies. **(D)** The 4,144 PLSDB plasmids predicted to encode multiple partition systems separated based on their partition system type combinations and whether they are predicted to have a circular (4,019/~97% of multiple partition system plasmids), linear (108/~3% of multiple partition system plasmids) or unknown (20/<1% of multiple partition system plasmids) topologies.

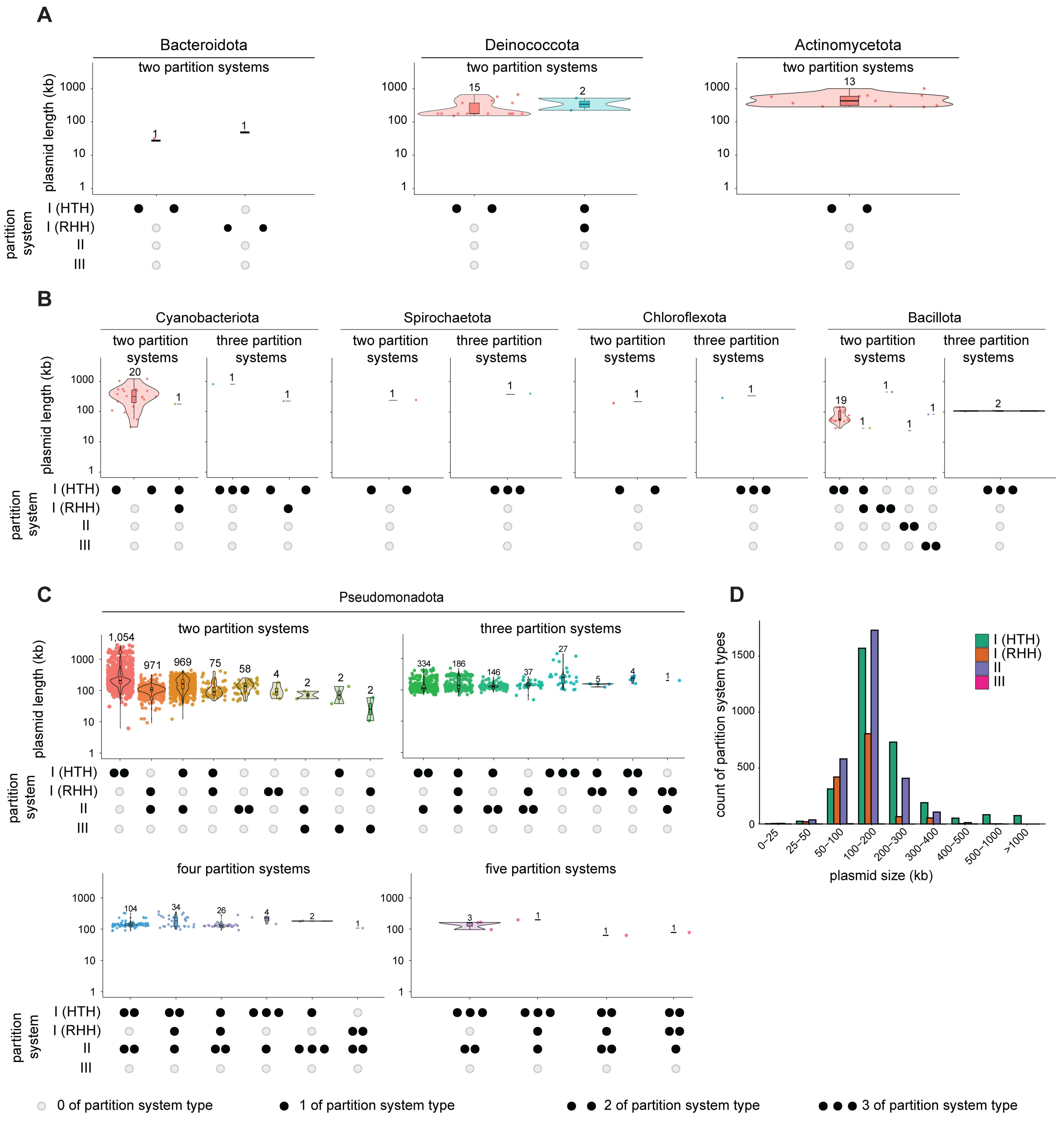

Supplementary Figure S4. Plasmids predicted to encode multiple partition systems were found across diverse bacterial phyla. **(A)** Plasmids isolated from the Bacteroidota, Deinococcota and Actinomycetota phyla were predicted to encode a maximum of two partition systems. **(B)** Plasmids isolated from the Cyanobacteriota, Spirochaetota, Chloroflexota and Bacillota phyla were predicted to encode a maximum of three partition systems. **(C)** Plasmids isolated from the Pseudomonadota phylum were predicted to encode two, three, four or five partition systems. Filled circles (single or multiple) indicate the presence of one or more corresponding partition system type(s), while unfilled circles indicate the absence of such partition system type(s). **(D)** Numbers of each plasmid partition system type found on the 4,144 PLSDB plasmids predicted to encode multiple partition systems, categorized by plasmid size. Each plasmid containing multiple partition systems contributes to the count for each system type it encodes (e.g. a plasmid predicted to encode both type-I (HTH-ParB) and type-I (RHH-ParB) systems contributes one count to each category).

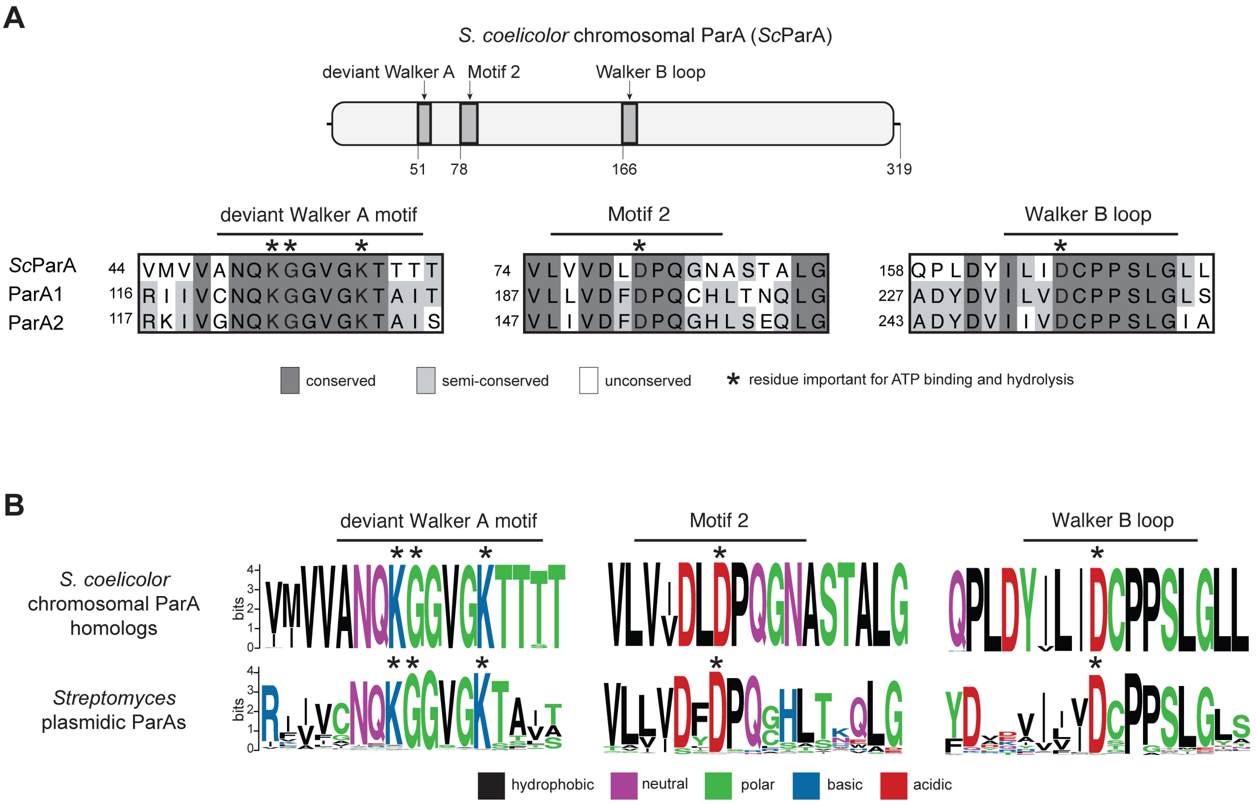

Supplementary Figure S5. Amino acid residues responsible for ATP-binding and hydrolysis are conserved in *Streptomyces* plasmid ParA proteins. **(A)** Top: domain structure of the *S. coelicolor* A3(2) chromosomal ParA protein (*Sc*ParA) with the deviant Walker A, motif 2 and Walker B loop highlighted. Bottom: multiple sequence alignment of the deviant Walker A motif, motif 2 and Walker B motif of *Sc*ParA, ParA1 and ParA2 show that the ATP-binding domain is highly conserved in ParA1 and ParA2. Asterisks denote functionally important residues for ATP binding and hydrolysis. **(B)** Sequence logos of ~3,500 *Sc*ParA homologs and the 156 *Streptomyces* plasmidic ParAs revealed that the deviant Walker A motif, motif 2 and Walker B motif are highly conserved in *Streptomyces* plasmidic ParAs (functionally important residues are highlighted with asterisks). Amino acids were colored based on their chemical properties (GSTYC, polar; QN, neutral; KRH, basic; DE, acidic; and AVLIPWFM, hydrophobic).

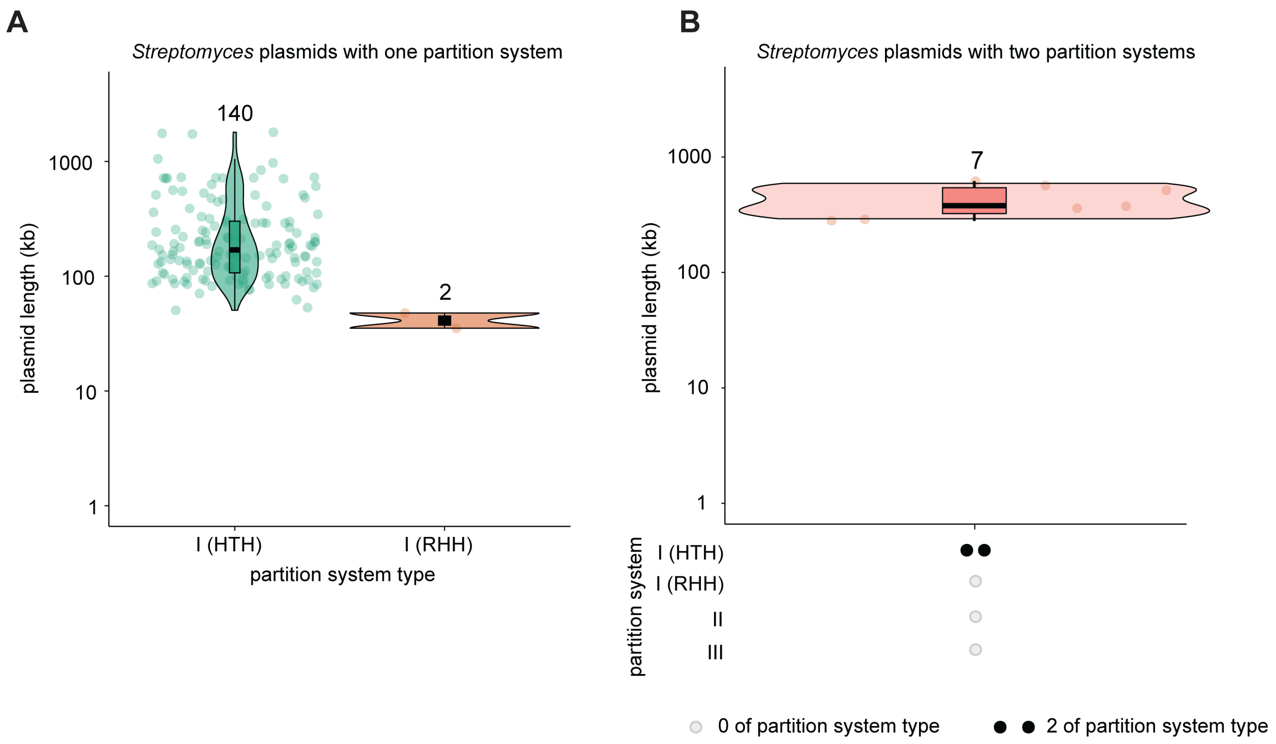

Supplementary Figure S6. *Streptomyces* plasmids were predicted to encode only type-I partition systems. **(A)** *Streptomyces* plasmids from the PLSDB with a single partition system exclusively encode type-I partition systems with the majority encoding type-I (HTH-ParB) partition systems. **(B)** *Streptomyces* plasmids from the PLSDB with multiple partition systems exclusively encoded dual type-I (HTH-ParB) partition systems. Filled circles (single or multiple) indicate the presence of one or more corresponding partition system type(s), while unfilled circles indicate the absence of such partition system type(s).

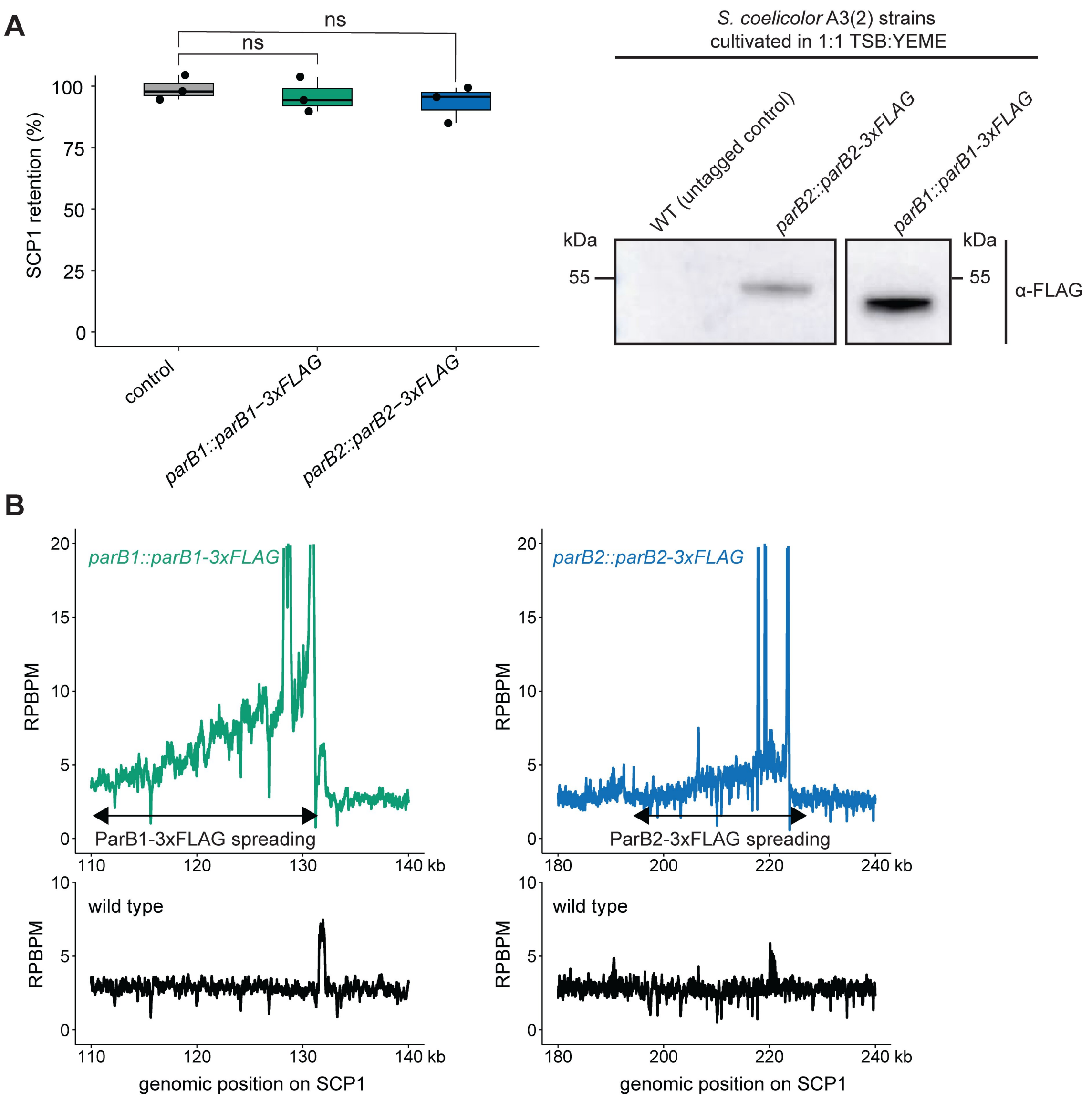

Supplementary Figure S7. ParB1-3xFLAG and ParB2-3xFLAG accumulated surrounding their cognate *parS* sites on SCP1. **(A) (left panel)** Insertion of a *3x-FLAG*-apramycin resistance cassette at the 3' ends of either the *parB1* or *parB2* genes did not disrupt SCP1 stability compared to a control strain (Δ*SCP1.94::apr*). Data was analyzed using a one-way ANOVA followed by Dunnett's multiple comparisons test (ns = not significant ($p \geq 0.05$)). **(right panel)** ParB1-3xFLAG and ParB2-3xFLAG proteins were produced during vegetative growth in liquid culture. α-FLAG immunoblot of 10 µg total cell lysates isolated from *S. coelicolor* A3(2) wild type (WT, untagged control), *S. coelicolor parB1::parB1-3xFLAG* and *S. coelicolor* A3(2) *parB2::parB2-3xFLAG* cultivated in 1:1 TSB:YEME liquid culture for 15 hours. **(B)** α-FLAG ChIP-seq profiles of *S. coelicolor* A3(2) *parB1::parB1-3xFLAG, parB2::parB2-3xFLAG*, and wild type show that ParB1-3xFLAG and ParB2-3xFLAG accumulated asymmetrically ~15-20 kb downstream of their cognate *parS* sites on SCP1. ChIP-seq profiles were plotted with the x-axis representing genomic position (kb) and the y-axis representing the number of reads per base pair per million (RPBPM). ChIP-seq was performed in biological duplicate and a representative profile is shown.

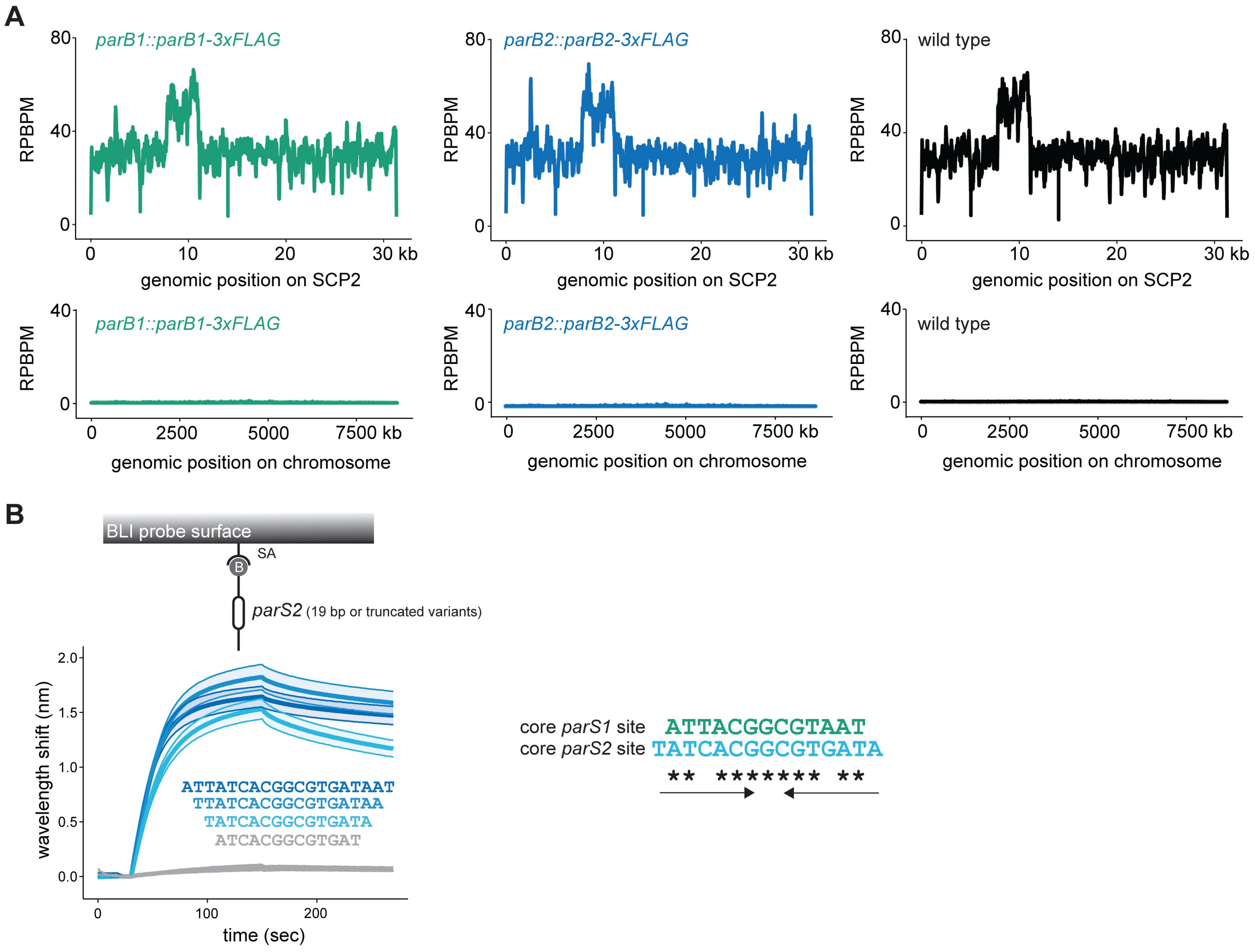

Supplementary Figure S8. ParB1-3xFLAG and ParB2-3xFLAG do not bind to the *S. coelicolor* A3(2) chromosome or SCP2. **(A)** α-FLAG ChIP-seq profiles of *S. coelicolor* A3(2) *parB1::parB1-3xFLAG*, *parB2::parB2-3xFLAG*, and wild type show no enrichment of ParB1-3xFLAG or ParB2-3xFLAG on the chromosome or SCP2. ChIP-seq profiles were plotted with the x-axis representing genomic position (kb) and the y-axis representing the number of reads per base pair per million (RPBPM). ChIP-seq was performed in biological duplicate and a representative profile is shown. **(B)** Left: BLI profiles of 1 µM ParB2 binding to a series of 50-bp DNA duplexes containing either the 19-bp *parS2* site identified from ChIP-seq experiments (dark blue) or truncated variants (gradient blue). The truncated *parS2* variant that ParB2 did not bind strongly to is colored in grey. Mean and standard deviation (shading) are shown for three replicates. Right: an alignment of the core *parS1* and *parS2* sites demonstrate a high level of sequence similarity between the two sites. Asterisks indicate conserved nucleotides. Convergent arrows indicate both *parS* sites are inverted repeats.

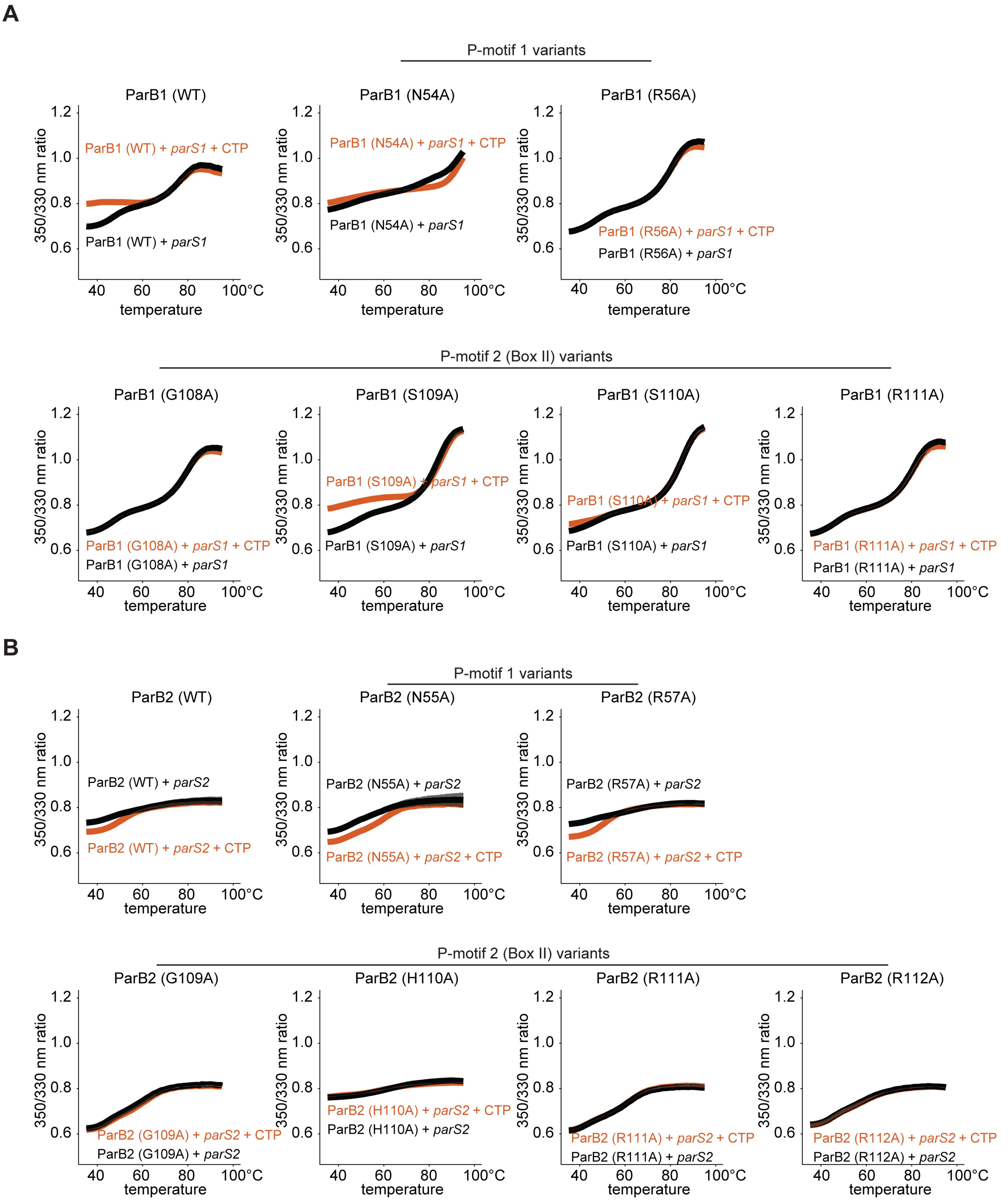

Supplementary Figure S9. Alanine mutagenesis of the CTPase domains of the SCP1 ParB proteins impaired CTP binding. **(A)** DSF unfolding profile of 4 µM ParB1 WT or ParB1 CTPase domain variants with 2 µM *parS1,* in the presence or absence of 1 mM CTP. **(B)** DSF unfolding profile of 4 µM ParB2 WT or ParB2 CTPase domain mutants with 2 µM *parS2,* in the presence or absence of 1 mM CTP. Mean and standard deviation (shading) from three replicates are shown.

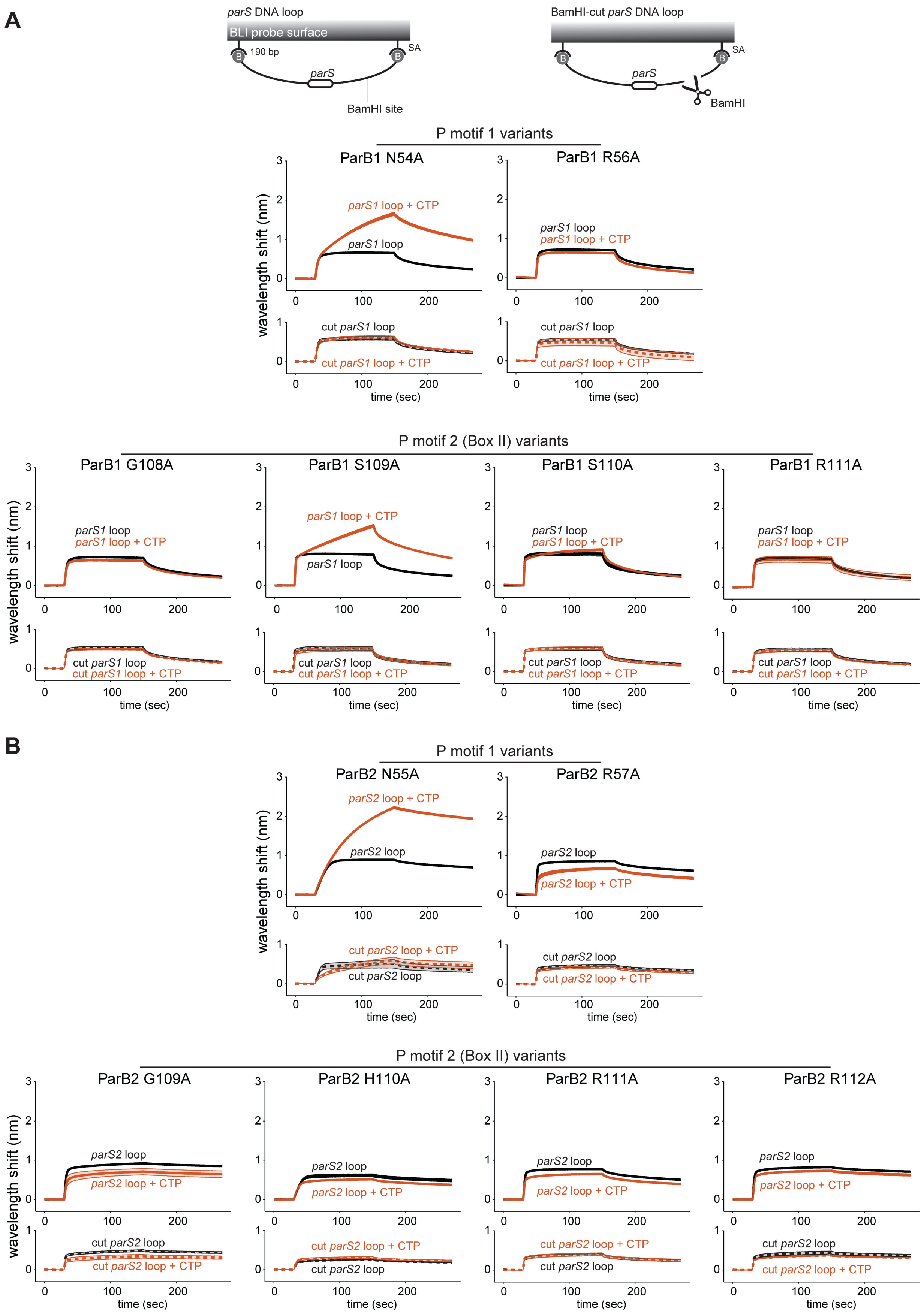

Supplementary Figure S10. Alanine mutagenesis of the CTPase domains of the SCP1 ParB proteins impaired the ability to accumulate on DNA *in vitro*. **(A)** BLI analysis of the interaction between 1 µM of ParB1 variants, in the presence or absence of CTP, with an intact or BamHI-restricted DNA loop. A 190-bp dual biotinylated *parS1*-containing DNA was attached to the streptavidin (SA) coated probe to create a closed DNA loop where both ends were blocked. The closed DNA loop was subsequently restricted by BamHI to create a free end (BamHI-cut DNA). **(B)** BLI analysis of the interaction between 1 µM of ParB2 variants, in the presence or absence of CTP, with an intact or BamHI-restricted DNA loop. The mean and standard deviation (shading) are shown for three replicates.

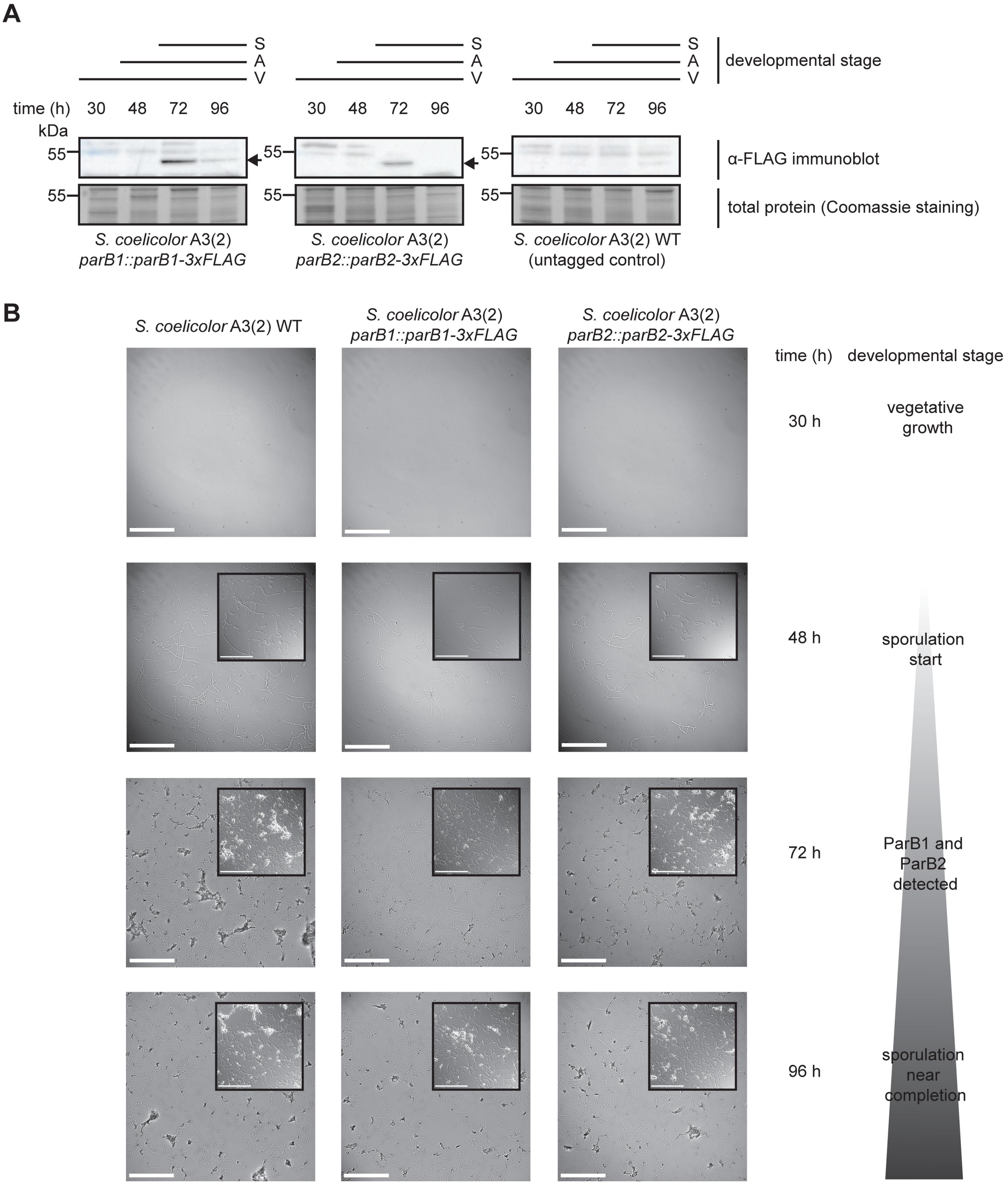

Supplementary Figure S11. ParB1-3xFLAG and ParB2-3xFLAG protein levels across developmental stages of *S. coelicolor* A3(2). (A) α-FLAG immunoblots of 30 µg total cell lysates isolated at the indicated timepoints from *S. coelicolor* A3(2) *parB1::parB1-3xFLAG, S. coelicolor* A3(2) *parB2::parB2-3xFLAG* and *S. coelicolor* A3(2) wild type (WT, untagged control), cultivated on cellophane discs overlaid on top of SFM agar. The timepoints, in hours (h), at which biomass were harvested, and the developmental stage (vegetative hyphal growth (V), aerial hyphal growth (A), and sporulation (S)) are shown, as judged by microscopic examination (see panel B). A separate Coomassie-stained SDS-PAGE gel of the same concentrations of each sample served as a loading control. Immunoblots were performed at least twice, and a representative immunoblot is shown. (**B)** Representative DIC images of coverslip impressions of *S. coelicolor* A3(2) wild type (WT), *S. coelicolor* A3(2) *parB1::parB1-3xFLAG* and *S. coelicolor* A3(2) *parB2::parB2-3xFLAG* strains grown on cellophane discs on SFM agar, as cultivated for the α-FLAG immunoblotting in panel A. Images were taken at the indicated timepoints (corresponding to the α-FLAG immunoblot timepoints in panel A). Only aerial hyphae undergoing sporulation or spores should attach to the hydrophobic coverslip. Spores were detected from the 48-hour timepoint in all strains. Insets show magnifications of the same region. Scale bar, 50 µm. All experiments were performed in triplicate, and a representative image is shown.

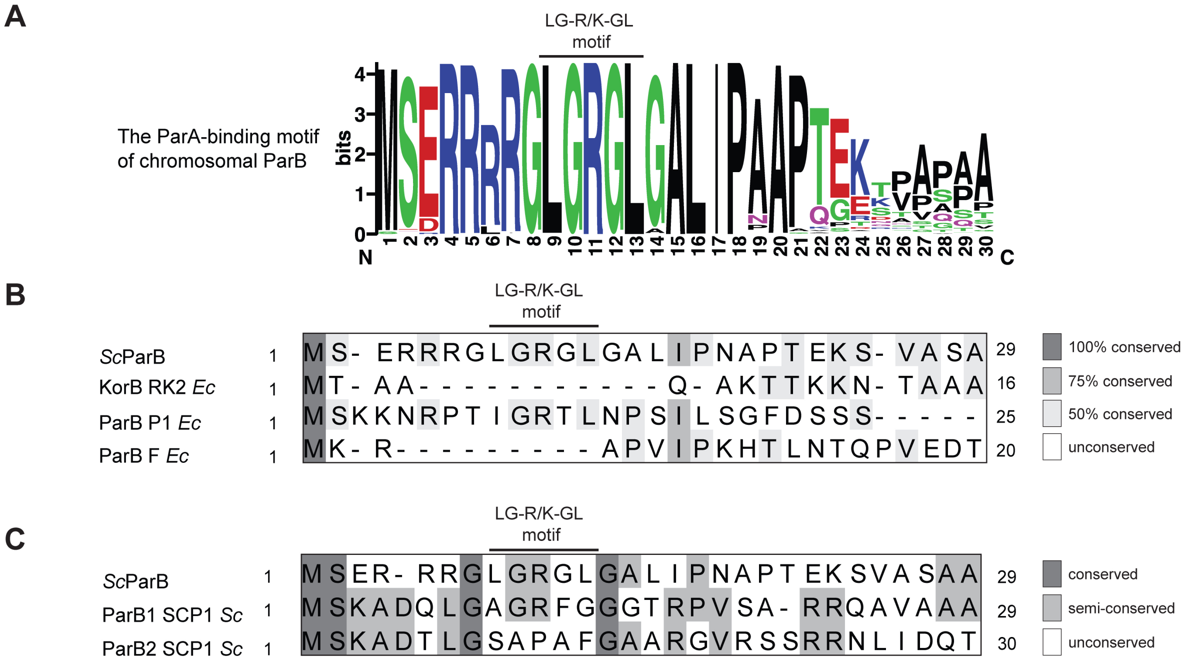

Supplementary Figure S12. The putative ParA-binding motifs of SCP1 ParB1 and ParB2 and *Sc*ParB are distinct. **(A)** Amino acid sequence alignments, shown as WebLogos, of ~3,500 ParB homologs obtained by pBLAST searches using *Sc*ParB as the query, reveals that the ParA-binding motif, which comprises the first 30 amino acids including the LG-RK-GL motif is highly conserved. **(B)** Multiple sequence alignments of the ParA-binding motif of *Sc*ParB with plasmid ParB proteins reveal that this motif is not conserved in plasmid ParB proteins. The proteins and their Uniprot accession numbers aligned are ParB from *S. coelicolor* (Q9RFM2), KorB from the RK2 plasmid of *E. coli* (P07674), ParB from the P1 plasmid of *E. coli* (P07621) and ParB (or SopB) from the F plasmid of *E. coli* (P62558)234. **(C)** Multiple sequence alignments of the putative ParA-binding motif of *Sc*ParB, SCP1 ParB1 and ParB2 reveal that this motif is also not conserved.
